# Differences in PD-L1 Expression between oral and oropharyngeal squamous cell carcinoma

**DOI:** 10.1371/journal.pone.0269136

**Published:** 2022-05-27

**Authors:** Sebastian Blatt, Maximilian Krüger, Constantin Rump, Stefanie Zimmer, Keyvan Sagheb, Julian Künzel

**Affiliations:** 1 Department of Oral and Maxillofacial Surgery, University Medical Center, Mainz, Germany; 2 Institute of Pathology and Tissue Bank, University Medical Center, Mainz, Germany; 3 Department of Otorhinolaryngology, University Hospital Regensburg, Regensburg, Germany; Hamad Medical Corporation, QATAR

## Abstract

Treatment of metastasized or recurrent oral (OSCC) and oropharyngeal (OPSCC) squamous cell carcinoma remains challenging. Targeted antibody-based therapy inter alia for PD-1 / PD-L1 axis shows promising results, but whether PD-L1 expression varies between the subentities remains unclear. The expression pattern of PD-L1 (EPR19759 antibody, Abcam, Berlin, Germany) and p16 (CINtech® Histology Kit, Ventana, Oro Valley, USA) was determined immunohistochemically and analyzed by HALO™ Image Analysis Software (Indica Lab, Albuquerque, USA). For PD-L1, combined positivity score (CPS), tumor proportion score (TPS) and histoscore, were assessed and results correlated with epidemiological data. In total, 161 patients (OSCC: n = 78, OPSCC: n = 83) were included. A mean of 43.6% (±34.0%) of the specimen showed increased PD-L1 expression that did not differ quantitatively between subentities (TPS: p = 0.159, CPS: p = 0.078), but qualitatively (histoscore: p = 0.003). In the mean follow-up period (45.6 months), contrary to age (p = 0.006) and advanced T-Status (p = 0.018), PD-L1 expression did not correlate with overall (OS, p = 0.191) and recurrence free survival (RFS: p = 0.193) in both subentities. No correlation of p16 and PD-L1 expression was found (p = 0.844). PD-L1 is differentially expressed between OSCC and OPSCC, however without influence on OS. Furthermore, p16 status was not related to PD-L1 expression. This may have implications for future (immune) therapeutical approaches for oral cancer.

## Introduction

Head and neck squamous cell carcinoma (HNSCC) and its subentities oral (OSCC) and oropharyngeal squamous cell carcinoma (OPSCC) represent a life threatening disease that causes around 160,000 deaths each year [1]. Among classical risk factors such as tobacco and alcohol consumption, infection with human papilloma virus (HPV), especially high risk subtypes HPV 16/18, contribute to carcinogenesis, primarily in OPSCC [2, 3]. About 45% of all OPSCC are associated with HPV infection [4]. The increasing number of OPSCC, despite the decreasing risk behavior of classical risk factors, is attributed to the increased number of HPV-induced tumors [5]. Despite great efforts in surgical and adjuvant treatment options, no significant increase in overall (OS) or recurrence free survival (RFS) could be achieved in recent years [6].

In this context, inhibition of checkpoint pathways of carcinogenesis has provided a new and promising approach in the direction of personalized medicine: Programmed cell death-1 (PD-1), an important inhibitory receptor, is critical for the maintenance of central and peripheral T cell tolerance [7]. However, PD-1, which is upregulated on activated and exhausted T cells, also limits productive immune responses against pathogenes and cancer cells. PD-1 signaling is induced upon binding of its ligands, PD-1 ligand-1 (PD-L1) and PD-1 ligand-2 (PD-L2). The expression of PD-L2 is mainly restricted to professional antigen presenting cells (APCs) like macrophages and dendritic cells (DCs), whereas PD-L1 is also expressed in non-hematopoietic tissues and can thus be regarded as the major PD-1 ligand. Importantly, PD-L1 is also upregulated in the tumor microenvironment and is found in a large variety of tumor cells. Tumor infiltrating lymphocytes frequently express PD-1, providing a rationale for therapeutically disrupting the PD-1/PD-L1 interaction to improve anti-tumor responses. Currently, several PD-1 and PD-L1 antibodies are in clinical use for the treatment of various solid cancers and lymphomas, and blocking of the PD-1 pathway is able to induce impressive response rates across a broad spectrum of tumor types, like metastasized and recurrent solid tumors such as lung cancer, melanoma and gastric cancer [8–10]. Here, a recent meta-analysis confirmed the superiority of checkpoint inhibitors over conventional chemotherapy in pretreated non-small-cell lung cancer patients [11]. Consequently, pembrolizumab, a PD-1 antibody, is now recommend in first line therapy option in non-curable, advanced metastasized and recurrent OSCC and OPSCC when PD-L1 is overexpressed [12]. Nivolumab is another antibody that has been approved by the FDA for the treatment of recurrent or metastatic HNSCC [13]. However, there is insufficient evidence and inhomogeneous data to which content these immunological axes are expressed. Up to date, there is no comparative study that evaluates PD/PD-L1 expression in the specific subentities of OSCC in comparison to OPSCC. Some studies see high expression rates of over 90% [14], whereas others demonstrate low values questioning the benefit of immunotherapy benefit [15]. Furthermore, there is an ongoing controversy if, and to what extent, PD-L1 overexpression influence overall (OS) and recurrence free survival (RFS) among these entities [16, 17]. Additionally, a correlation between PD-L1 expression and HPV infection, measured by the surrogate marker cyclin-dependent kinase inhibitor 2A oncoprotein (p16) expression, is hypothesized [18, 19]. Since the prevalence of HPV infection is recognized much lower in the oral cavity, this study aims to investigate expression pattern of PD-L1 in p16 negative and positive oral and oropharyngeal carcinoma and analyze possible implications for patient outcome. Therefore, this study is the first to provide comparative evidence for the respective subentities of HNSCC that may help to refine immunotherapeutic approaches in the clinical setting. Here, PD-L1 and p16 expression pattern was assessed for OSCC and OPSCC respectively and correlated with epidemiological features of the patients such as OS or DFS.

## Materials and methods

### Patients collective

Patients with surgically treated OSCC or OPSCC (n = 161) of the Department of Otorhinolaryngology and the Department of Oral and Maxillofacial Surgery – Plastic Surgery, University Medical Centre, Mainz Germany, were included in the study. Informed consent was obtained from all individuals participated in the study. The analysis was performed within the Declaration of Helsinki and its later amendments and the approval of the local ethic committee (“Bezirksärztekammer Rheinland Pfalz”, nr 2018-13844). All participants gave informed consent.

All patients received primary surgical tumor therapy with or without adjuvant therapy in accordance with recommendation of the interdisciplinary tumor conference recommendation and existing guidelines. Age, gender, risk behavior, TNM classification (7^th^ Edition), tumor localization, OS, RFS, PD-L1 expression and HPV p16 status were analyzed for all patients. Distant metastasis at the time point of initial staging that led to a primarily systemic therapy was defined as exclusion criteria.

### PD-L1 expression

PD-L1 expression was analyzed by immunohistochemical staining of tissue microarrays (TMAs) of the respective primary tumors as previously described [20]. Briefly, to create TMAs, tissue punches were taken from the tumors, placed in paraffin blocks, and cut into thin layers before transferred to a microscope slide. Next, TMAs were stained in the CoverStainer (Dako/Agilent, Santa Clara, USA) with PD-L1 antibodies. According to the standards of the Department of Pathology, University Medical Centre Mainz, Germany, the anti-PD-L1 rabbit antibody EPR19759 (diluted 1: 250, Abcam, Berlin, Germany) was used. Tissue samples were provided by the tissue bank of the University Medical Center Mainz in accordance with the regulation of the tissue biobank and the approval of the ethics committee of University Medical Center Mainz (Project number: #GB000218).

### p16 expression

The expression of the HPV surrogate marker protein p16 was examined immunohistochemically as previously described [21, 22]. Here, anti- p16INK4a (E6H4) mouse antibody (CINtech ® Histology Kit Ventana, Oro Valley, USA) was used and immunohistochemistry was carried out analogously to the PD-L1 staining according to the standards of Department of Pathology, University Medical Centre Mainz, Germany. In accordance to the HNSCC guideline by the Federal Association of German Pathologists, tumors with more than 70% stained cells were assessed as p16 positive [23].

### Technical evaluation

**Fig 1:**
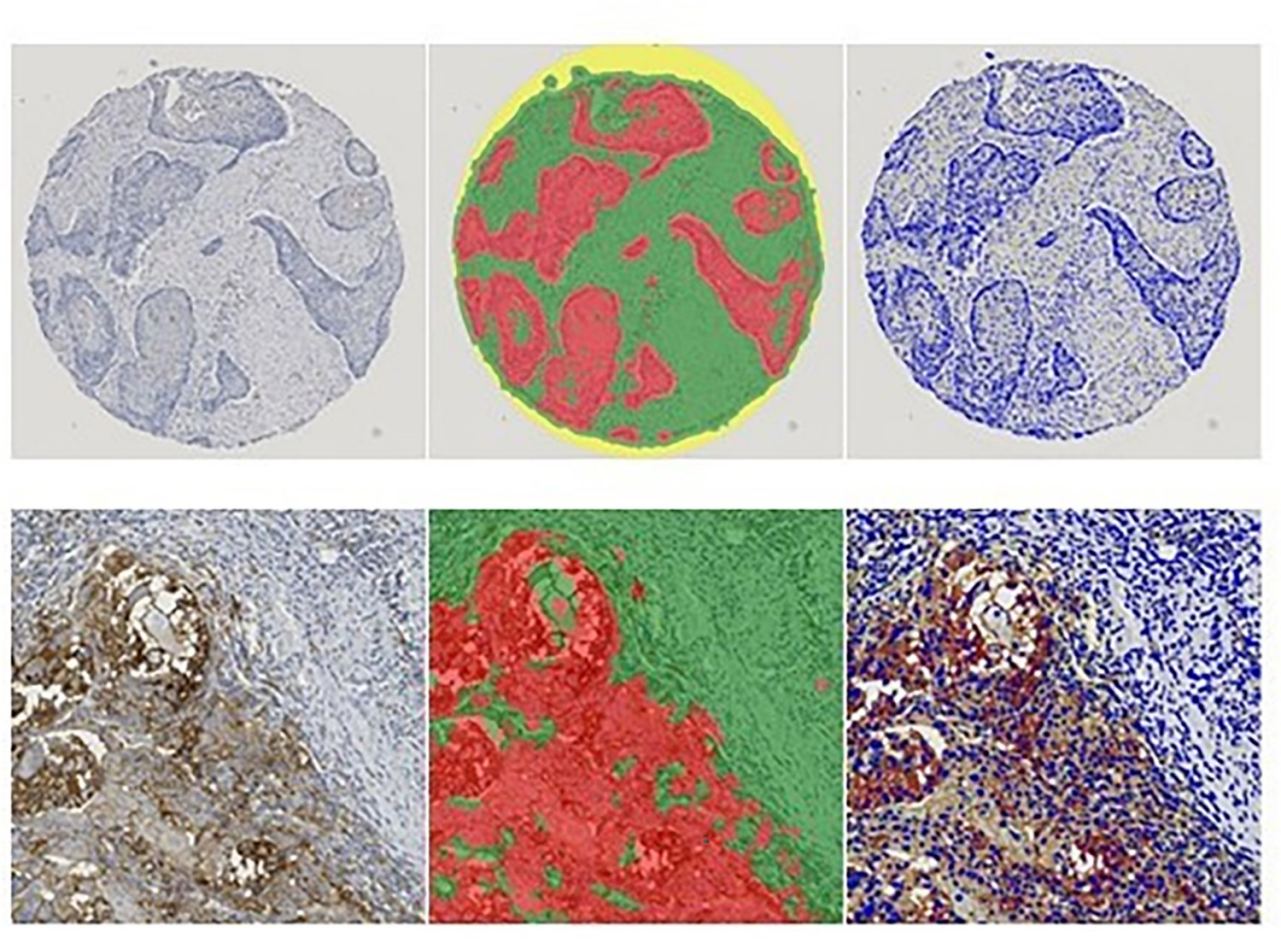
Top row: Representation of the complete core. Bottom row: section of the core, left: PD-L1 staining(20 fold magnification). Middle: Classifier shows stromal cells (green), tumor cells (red), microscope slides (yellow). Right: The computer-aided analysis counts the cells involved based on the cell nuclei (dark blue) and differentiates the colored cells based on the membrane staining, subdivided according to intensity (yellow, orange, red).

The stained tumor tissue samples were evaluated blindly by a pathologist using the HALO™ Image Analysis Software (Indica Lab, Albuquerque, USA). Combined Positivity Score (CPS) and Tumor Proportion Score (TPS) as quantitative analyses of expression were achieved. The CPS is defined as all (PD-L1)-stained cells / viable tumor cells x 100 [12]. The TPS refers to the percentage of all tumor cells that have accumulated the antibody’s dye in their cell membrane [24]. The software enabled a semi-quantitative assessment of the staining intensity (weak, medium and strong, Fig 1). In addition, the histoscore (H-score) as a qualitative assessment from this semiquantitative immunohistochemical staining was calculated as previously described [25]: Histoscore (values from 0 to a maximum of 300 in total numbers) = 1x% weakly stained cells + 2x% medium stained cells + 3x% strongly stained cells.

### Statistical analysis

Statistical analysis was performed using SPSS version 23 (IBM Deutschland GmbH, Ehningen, Gemany). Results were displayed in mean with its standard error of mean (SEM). Kaplan-Meier graphs with log rank test and cox regression analysis were performed to analyze OS and RFS and possible associations. Differences between the groups were compared using the Mann-Whitney U test since non normal distribution was detected via Shapiro-Wilk-Test. Pearson’s chi-square was used to examine correlations between variables (nominal / ordinal). As conventional, a p-value ≤ 0.05 was set to be significant.

## Results and discussion

### Epidemiological features of the patient collective

A total of 161 patients were included in the study (Table 1). The mean age was 64.4 (±10.6) years (♂: n = 99, 61.5%, ♀: n = 62, 38.5%). The collective was divided in two groups, depending on the manifest subentity (OSCC: n = 78, OPSCC: n = 83). Classical risk factors were strongly represented throughout the collective (87.0% with a positive anamnesis in total), of which 72.0% were smokers, 70.8% admitted to drinking alcohol. The risk behavior was significantly more pronounced in patients with OPSCC vs. OSCC (95.2% vs. 78.2%, p = 0.002). In OSCC, the tongue (28.2%) and the floor of the mouth (25.6%) were the most common locations. For OPSCC, majority of tumors was found in the palatine tonsils (33.7%), 28.9% were located at the soft palate. Most of the tumors were diagnosed at an early stage (T1/2, 67.1%) in contrast to advanced tumor stages of one third at the time of diagnosis (T3/4, 32.9%). Initially, lymph nodes were already involved in half of the patients (N0 = 53.4%, N+ = 50%). Most of the tumors were well differentiated (G1/2, 66.0%).

**Table 1:** Epidemiological features of the patients cohort (n = 161).

	Total	In %
Age Group		55.9
		44.1
Gender	Male (n = 99)	61.9
	Female (n = 62)	38.1
Risk factors	Smokers (n = 116)	72.5
	Non-smokers (n = 45)	27.5
	Alcohol (n = 114)	71.2
	No alcohol (n = 47)	28.8
T status	T1(n = 48)	29.8
	T2 (n = 60)	37.3
	T3 (n = 30)	18.6
	T4 (n = 23)	14.3
N Status	0 (n = 86)	53.4
	1 (n = 19)	11.8
	2a (n = 9)	5.6
	2b (n = 30)	18.6
	2c (n = 13)	8.1
	3 (n = 4)	2.5
Tumor margins	R0 (n = 117)	70.7
	R1 (n = 44)	29.3
Entities	OSCC (n = 78)	48.5
	OPSCC (n = 83)	51.5
Tumorsite	Buccal mucosa (n = 10)	6.2
	Floor or the mouth (n = 20)	12.4
	Tongue (n = 22)	13.7
	Maxilla (n = 9)	5.6
	Mandibula (n = 17)	10.6
	Palatine tonsile (n = 28)	17.4
	Tongue base (n = 19)	11.8
	Lower oropharynx (n = 12)	7.5
	Soft palate & uvula (n = 24)	14.9

**Fig 2:**
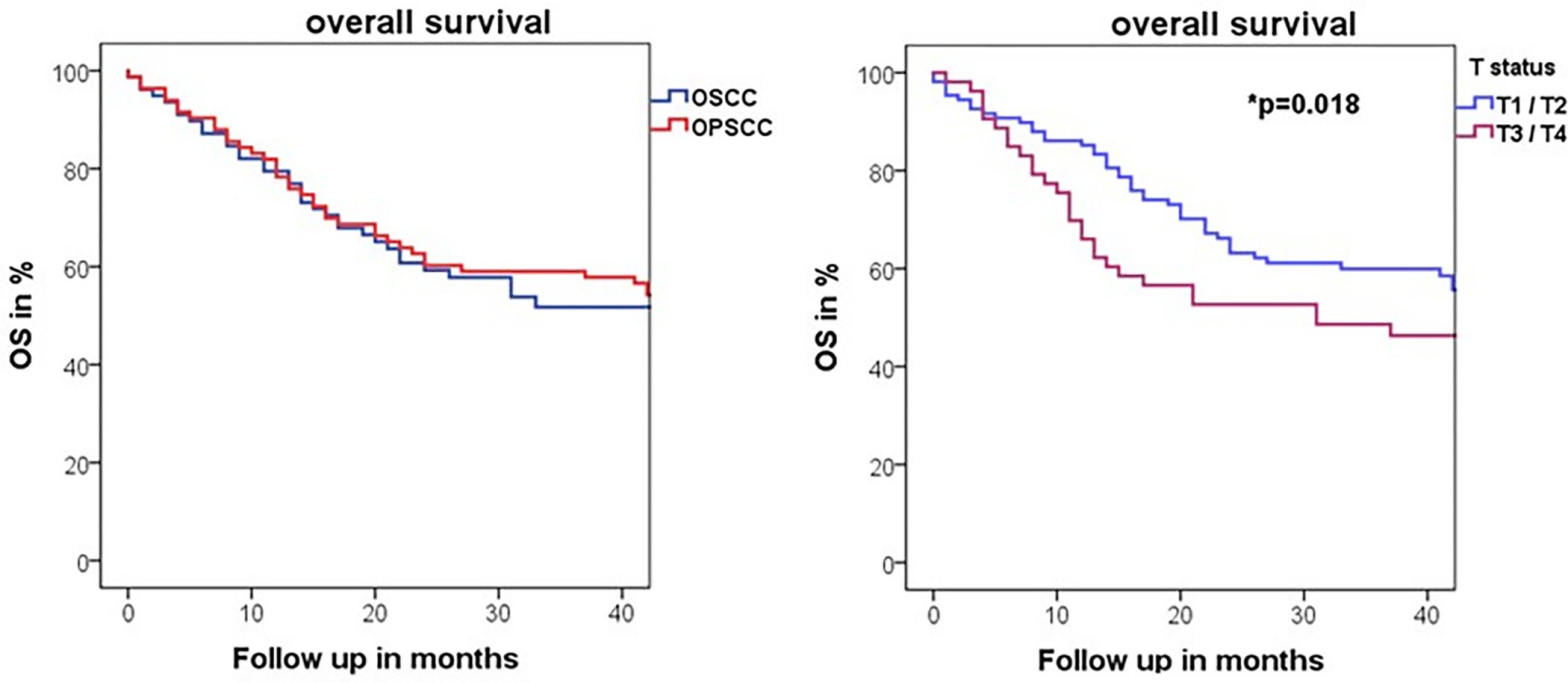
Kaplan-Meier curve illustrating A) Overall survival (OS) in respective of HNSCC subentity (OSCC blue, OPSCC red, p = 0.379) and B) significant influence of advanced T-Status on OS in both entities (T1/2 (blue) vs. T3/4 (purple), p = 0.011).

Of the 161 patients in this study, 73 had died within the median follow-up of 45 months (47.4%). The Kaplan-Meier analysis showed overall no difference for OS between OSCC and OPSCC (p = 0.448). Log Rank test found advanced T status as an independent predictor for reduced OS in both entities (T1/2: 93.9 months vs. T3/4: 54.7 months, p = 0.018, Fig 2A, 2B). In multivariate analysis, higher age (over 65 years) showed a significant influence on OS (p = 0.006).

### PD-L1 expression profile

**Fig 3:**
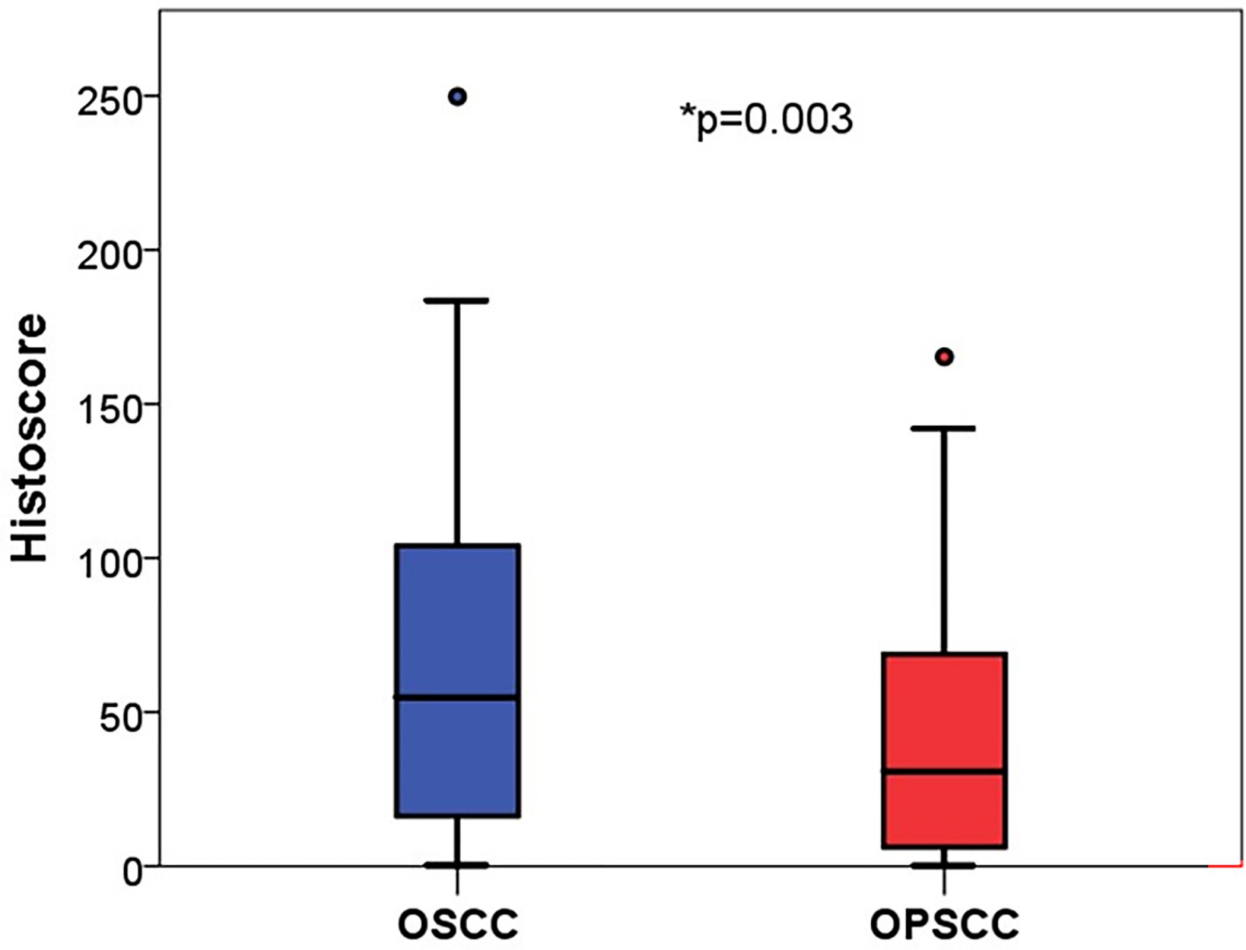
Assessed via histoscore, OSCC (blue) and OPSCC (red) expressed PD-L1 qualitatively significant different (mean values: 47.8 vs. 31.0, * p = 0.003).

For all samples, an average of 43.6% of the tumor cells demonstrated positive PD-L1 expression. The TPS settled between a minimum of 0% and a maximum of 98.88%. Comparing both subentities, the mean TPS for OSCC was 47.8% whereas 39.6% for OPSCC (p = 0.159). However, in qualitative assessment, OSCC displayed a significant higher histoscore in comparison to OPSCC (Fig 3, 65.4 vs. 41.2 p = 0.003). The CPS had a range from 0.5 as the lowest value up to a defined upper limit of 100 as the maximum [26]. The mean value was 66.35. The value for OSCC was higher than for patients with an OPSCC without reaching statistical significance (OSCC: 70.57 vs. OPSCC: 62.39, p = 0.078). No influence of early tumor stages (T1/2) versus late tumor stages (T3/4) on the expression pattern of PD-L1 could be observed (TPS: p = 0.483; CPS: p = 0.359; Histoscore: p = 0.429). Furthermore, grading had no influence on PD-L1 expression profile. In addition, no association of PD-L1 expression and patient outcome could be found. To assess a possible cut off value, it could be demonstrated that patients with a TPS> 50% of PD-L1 expression tented to a higher OS on average (p = 0.191).

### p16 expression profile

Overall, 37 tumors (23%) displayed a positive p16 expression. In comparison to OSCC, statistically significant more OPSCC were found p16 positive (16.7% vs. 28.9%, p = 0.048). Within the OPSCC collective, subanalysis showed that tumors of the palatine tonsil were significantly more often associated with a positive p16-status than any other anatomical localization (p = 0.012). Overall, there was no association between the HPV + or HPV- OPSCC and OSCC and PD-L1 expression pattern (all tumors: p = 0.844, OSCC, p = 0.507; OPSCC, 0.433). A positive p16 status had a significant impact on OS of OPSCC (p = 0.016) and tented to influence RFS (p = 0.032) in comparison to HPV- OPSCC. For OSCC, no further association to OS and/or RFS could be found.

The aim of this study was to analyze PD-L1 expression pattern of OSCC and OPSCC comparatively and to detect possible correlations with p16 status as well as epidemiological features such as overall- und recurrence free survival. In the current literature, the PD-L1 expression revealed a wide range and reached from the lowest percentage of 9.5% [15] to the highest percentage of 94% [14] in both entities. Up to date, no single study could be obtained that distinguished analyzed the expression pattern of PD-L1 between the subentities OSCC and OPSCC. As a major result of this study, analysis of CPS and TPS revealed a tendency to a differentially expressed quantitative PD-L1 profile in OSCC and OPSCC. This result is in accordance with a recent meta-analysis on PD-L1 expression in HNSCC [27].

In addition, major drawback of quantitative analysis of PD-L1 expression consists in the fact that an optimal cutoff seems not clear yet to distinguish if an immune-therapeutically approach may be feasible. This becomes all the more important as immune therapy can cause causes a variety of immune-related adverse events that may ultimately attribute to patients’ death [28].

The rating of PD-L1-postivity in the current studies followed either a binary classification (expressed or not) or semiquantitative from 0 to 2 [16, 29] or 3 [30] definition. The positivity is defined within the scope from > 0 [14, 16, 17, 31–35] up to >20% [36, 37] of the investigated tumor cells. Some studies worked with three cut-offs for 1%, 5% and 10% [38, 39] whereas other did not distinguish between weak staining and no staining overall [16, 17, 40]. In many studies, a cut-off TPS ≥ 50% is used [8]. If applied in this study, about half of the OSCC and nearly 40% of the OPSCC would be considered as PD-L1 positive.

In a consensus paper, the Society for Immunotherapy of Cancer has set a cut-off for TPS of ≥1% for carcinomas of the head and neck area [24]. In this case, 98.8% of the tumors in this study would be positive for PD-L1. Additionally, a limit value for CPS ≥1 was selected in the mentioned consensus paper. When applied in the presented collective, only 2 cases (1.25%) would not be positive for PD-L1. These limit values do not seem to be suitable to provide the necessary selectivity for analysis of the expression pattern before considering immunotherapeutically therapy regimens. Therefore, other studies commit themselves to a TPS cut-off of 10% in their work that correlated with a decreased OS [41].

Furthermore, controversies exist about the implemented antibodies to detect PD-L1 expression. In this context, a comparison of antibodies was found in two studies showing that the PD-L1 status can be influenced by the choice of assay [32, 39]. Prospective clinical trials are much in need to further evaluate these challenges.

Based on the results obtained in this study, PD-L1 expression was not seen a prognostic factor for OS and RFS. Neither a very high nor a very low level of PD-L1 had an influence on OS. Opposing statements can be found in the studies of Ahn et al. and Hanna et al. [29, 42]. In their studies, a high expression of ≥ 50% (based on membranous staining intensity and > 10% of tumor cells with strong positivity respectively) of PD-L1 showed favorable outcome. In contrast, in the study by Mueller et al. a strong correlation was observed between high PD-L1 expression and a worse OS [16]. Karpathiou et al. showed in their study that a high PD-L1 expression is associated with a slightly improved OS [17]. In summary, there is contrarily evidence about the prognostic value of PD-L1 expression in HNSCC.

Having this limitation in mind, one may hypothesize that a qualitative assessment of PD-L1 expression may be more efficient to detect possible responsiveness to immunotherapy in comparison to quantitative assessment. Here, a significant difference between the two sub-entities in the histoscore as a tool to assess these qualitative differences was found. This suggests that the OSCCs express PD-L1 with a higher intensity and tent to do so more frequently in comparison to OPSCC. Unfortunately, there are few comparative studies to compare these findings as the histoscore obtained by immunohistochemistry has not received much attention so far. Sadeghi et al. also used the H-score in their study on CD44 in colorectal cancer and divided their collective into two subgroups based on the mean value [43]. Others show h-score to determine cancer steam cell marker inter alia for breast cancer [44]. In this context, Mueller et al. have seen strong staining associated with more aggressive tumor growth [16]. For clinical application, this study allows the conclusion that not only the quantitative expression of PD-L1 should be examined, but also the qualitative staining intensity with the help of the H-score.

The epidemiological characteristic of the investigated study cohort showed frequent localizations consistent with the literature: the tongue as the primary anatomical region for OSCC [45] and the palatine tonsil for OPSCC respectively [46]. In this study, a positive p16 status was found more frequently in OPSCC than in OSCC. These observations coincide with several comparable studies [47, 48]. It was shown that HPV associated tumorigenesis is more prevalent in OPSCC than in OSCC [3, 48]. However, contrary to the studies by Balermpas et al. [18] and Lyford-Pike et al. [19] there was no correlation between HPV positivity and PD-L1 expression in this study. Possible immunological interactions should be further evaluated to detect a potential role of viral oncogenesis and immune escape of OSCC and OPSCC.

There are certain shortcomings in this study. First and overall, the retrospective manner of this study may implicate a recall bias. As a strong example, the patients record were gathered from 2011 to 2017 and the 8^th^ TNM classification has not yet been applied consequently. Next, an unusual high number of early stage tumors were included in this study that may have an impact on survival statistics in comparison to other studies. Methodologically, IHC staining was evaluated using a software, but the program had to be adapted to the tumor by a blinded pathologist so that complete objectivity cannot be given. In addition, the HPV association was detected with p16 staining which is used as the IHC gold standard. However, it is only considered as a surrogate marker with certain shortcomings in comparison to PCR detection of the specific HPV phenotype [3]. Lastly, the presented study only focused on the evaluation of PD-L1 expression of the respective tumor cells. However, there is emerging evidence if immune cells rather than tumor cells should be addressed for assessment of PD-L1 expression as an useful prognostic biomarker in patients with oral cancer. Here, a study found overexpression of p16 and PD-L1 on immune cells of OPSCC to correlate with favorable patient’s outcome [49]. These findings need to be addressed in future prospective studies.

## Conclusion

In this study, no influence of PD-L1 expression on OS could be found. It was noticeable that the PD-L1 expression was 17.7% higher in OSCC than in OPSCC. This observation was not significant. A relationship between HPV and PD-L1 has not been established. It was found that OPSCC are significantly more likely to be p16+ than tumors of the oral cavity. Further studies are needed to investigate the role of PD-L1 in HNSCC.

## Supporting information

S1 Table (PDF)
