# Supplementary material for: Differences in PD-L1 Expression between oral and oropharyngeal squamous cell carcinoma
Source: PLoS One. 2022 May 27;17(5):e0269136. doi: 10.1371/journal.pone.0269136 (PMC9140279; doi:10.1371/journal.pone.0269136)
Supplement: S1 Table — (PDF) [file pone.0269136.s001.pdf]

ID	age	sex	smoker	alcohol	T	N	M	G	R	HPV	entity	localisation	TPS	CPS	H-score	OS	DFS	recurrence	death
1	76	1			1	0	0		0	0	OSCC	tongue	9,92	41,64	13,74	17,00	17,00	0	0
2	67	1	0	1	1	0	0		0	0	OSCC	tongue	88,44	171,68	54,50	35,00	35,00	0	0
3	67	1	0	0	2	2	0		0	0	OSCC	mandibula	83,66	426,00	18,94	35,00	35,00	0	0
4	70	2	0	1	4	0	0		0	0	OSCC	tongue	10,55	14,83	4,28	5,00	5,00	0	1
5	68	1	0	1	4	1	0		0	0	OSCC	mandibula	44,45	66,85	72,03	65,00	65,00	0	1
6	46	1	1	1	1	0	0	1	0	0	OSCC	tongue	88,71	110,56	31,59	27,00	27,00	0	0
7	51	2	1	0	1	0	0	1	0	0	OSCC	tongue	8,23	23,76	4,92	49,00	49,00	0	0
8	62	2	1	1	2	0	0	1	0	0	OSCC	oral mucosa	98,14	125,25	131,76	43,00	43,00	0	0
9	65	2	1	0	1	0	0	1	0	0	OSCC	oral mucosa	94,91	276,97	74,21	26,00	23,00	1	0
10	54	1	1	1	1	0	0	2	0	0	OSCC	tongue	89,61	152,22	1,80	45,00	45,00	0	0
11	63	1	1	1	2	0	0	2	0	0	OSCC	floor of mouth	92,80	265,13	28,57	28,00	27,00	1	0
12	60	1	1	1	2	0	0	2	0	0	OSCC	maxilla	12,92	19,43	31,89	24,00	24,00	0	1
13	62	2	0	1	1	0	0	2	0	0	OSCC	mandibula	8,17	63,88	55,10	38,00	38,00	0	0
14	45	2	1	0	2	0	0	2	0	0	OSCC	floor of mouth	94,49	118,00	6,44	36,00	36,00	0	0
15	61	2	0	1	1	0	0	2	0	0	OSCC	maxilla	88,73	178,04	118,61	43,00	43,00	0	0
16	60	2	0	1	2	0	0	2	0	0	OSCC	tongue	94,21	47,80	141,72	22,00	4,00	1	1
17	85	1	0	0	2	0	0	2	0	0	OSCC	oral mucosa	85,77	159,28	178,56	1,00	1,00	0	1
18	75	1	1	1	2	0	0	2	0	0	OSCC	floor of mouth	61,68	115,66	102,43	42,00	42,00	0	0
19	68	1	1	1	1	0	0	2	0	0	OSCC	oral mucosa	6,45	30,16	15,75	13,00	13,00	0	1
20	78	1	1	1	2	0	0	2	0	0	OSCC	floor of mouth	88,28	133,86	56,36	14,00	14,00	0	1
21	77	2	0	0	1	0	0	2	0	0	OSCC	tongue	5,67	9,87	91,22	27,00	27,00	0	0
22	78	2	0	0	1	0	0	2	0	0	OSCC	oral mucosa	89,86	137,22	51,89	38,00	31,00	1	0
23	88	2	1	1	2	0	0	2	0	0	OSCC	maxilla	56,30	105,35	45,28	84,00	84,00	0	1
24	70	2	1	0	2	0	0	2	0	0	OSCC	oral mucosa	2,84	12,28	142,73	36,00	36,00	0	0
25	76	2	1	0	1	0	0	2	0	0	OSCC	tongue	39,44	124,20	56,42	38,00	38,00	0	0
26	79	2	0	1	1	0	0	2	0	0	OSCC	mandibula	46,58	110,09	69,90	17,00	17,00	0	1
27	38	1	0	0	2	3	0	2	0	0	OSCC	tongue	5,91	28,99	44,33	27,00	13,00	1	0
28	91	1	0	1	2	1	0	2	0	0	OSCC	tongue	64,25	96,01	6,95	27,00	27,00	0	0

29	79	1	1	1	1	1	0	2	0	0	OSCC	maxilla	1,12	3,12	90,46	44,00	44,00	0	0
30	67	1	1	1	2	2	0	2	0	0	OSCC	mandibula	20,82	115,09	52,68	39,00	39,00	0	0
31	67	2	0	0	1	2	0	2	0	0	OSCC	tongue	53,77	57,81	43,44	8,00	8,00	0	1
32	61	1	1	1	4	0	0	2	0	0	OSCC	mandibula	86,29	127,44	13,42	41,00	41,00	0	0
33	49	1	0	1	4	1	0	2	0	0	OSCC	mandibula	34,37	144,33	19,96	31,00	31,00	0	1
34	65	1	1	1	3	2	0	2	0	0	OSCC	floor of mouth	28,33	29,47	1,18	21,00	21,00	0	1
35	63	2	0	0	4	2	0	2	0	0	OSCC	maxilla	70,42	97,67	45,92	13,00	13,00	0	1
36	80	1	1	1	4	2	0	2	0	0	OSCC	mandibula	87,64	142,05	122,20	11,00	13,00	1	1
37	76	2	0	1	4	2	0	2	0	0	OSCC	mandibula	16,92	25,50	118,62	42,00	42,00	0	0
38	68	2	1	1	3	2	0	2	0	0	OSCC	floor of mouth	56,60	129,50	183,61	11,00	6,00	1	1
39	61	1	1	1	2	0	0	3	0	0	OSCC	floor of mouth	75,94	183,78	16,27	17,00	17,00	0	0
40	46	2	0	1	2	0	0	3	0	0	OSCC	tongue	63,75	412,00	96,80	20,00	20,00	0	0
41	51	1	1	1	2	2	0	3	0	0	OSCC	floor of mouth	75,11	85,80	71,36	22,00	22,00	0	1
42	84	1	0	1	2	1	0	3	0	0	OSCC	tongue	41,93	49,67	43,07	9,00	9,00	0	1
43	77	1	1	1	2	2	0	3	0	0	OSCC	floor of mouth	27,68	56,73	57,58	26,00	26,00	0	1
44	82	2	0	0	2	3	0	3	0	0	OSCC	mandibula	5,19	14,58	128,51	,00	,00	0	1
45	58	1	0	1	3	0	0	3	0	0	OSCC	floor of mouth	2,99	6,31	15,78	104,00	59,00	1	0
46	49	1	1	1	4	1	0	3	0	0	OSCC	mandibula	53,27	119,63	106,07	35,00	35,00	0	0
47	54	1	1	1	3	1	0	3	0	0	OSCC	floor of mouth	2,30	24,20	11,27	4,00	4,00	0	1
48	62	1	1	1	4	2	0	3	0	0	OSCC	mandibula	29,06	159,01	129,79	6,00	6,00	0	1
49	65	2	0	0	3	2	0	3	0	0	OSCC	tongue	2,31	72,71	16,06	35,00	35,00	0	0
50	75	2	0	1	4	3	0	3	0	0	OSCC	mandibula	93,02	286,95	136,95	19,00	19,00	0	0
51	65	1	1	1	2	2	0	4	0	0	OSCC	floor of mouth	4,94	12,00	5,99	20,00	20,00	0	1
52	78	2	0	0	1	0	0		1	0	OSCC	maxilla	1,27	3,94	159,29	16,00	16,00	0	0
53	74	1	1	0	3	0	0	1	1	0	OSCC	tongue	45,72	50,38	20,35	9,00	114,00	0	1
54	79	1	0	0	4	2	0	1	1	0	OSCC	floor of mouth	51,24	221,97	45,29	15,00	15,00	0	1
55	58	1	1	1	1	0	0	2	1	0	OSCC	oral mucosa	5,61	21,50	91,33	26,00	26,00	0	0
56	67	1	1	1	1	0	0	2	1	0	OSCC	floor of mouth	63,47	106,67	149,93	19,00	14,00	1	1
57	90	2	0	1	2	0	0	2	1	0	OSCC	mandibula	47,47	144,03	21,62	2,00	2,00	0	1
58	54	1	1	1	2	2	0	2	1	0	OSCC	tongue	13,78	19,83	13,84	16,00	16,00	0	1

59	60	2	1	0	2	1	0	2	1	0	OSCC	floor of mouth	,31	,59	,29	125,00	7,00	1	0
60	64	1	1	1	4	0	0	2	1	0	OSCC	mandibula	16,35	112,25	56,69	43,00	43,00	0	0
61	75	1	0	0	4	0	0	2	1	0	OSCC	floor of mouth	85,43	93,45	77,16	14,00	14,00	0	1
62	80	1	1	0	3	0	0	2	1	0	OSCC	tongue	90,34	240,38	16,45	8,00	8,00	0	1
63	70	2	1	1	3	0	0	2	1	0	OSCC	floor of mouth	77,08	94,23	104,55	17,00	17,00	0	1
64	56	1	1	1	4	1	0	2	1	0	OSCC	floor of mouth	92,73	104,63	115,39	33,00	33,00	0	0
65	85	2	1	0	4	2	0	2	1	0	OSCC	mandibula	90,17	189,56	103,96	1,00	1,00	0	1
66	87	1	1	1	4	2	0		0	1	OSCC	mandibula	7,14	22,34	30,10	6,00	6,00	0	1
67	83	1	1	1	1	0	0	1	0	1	OSCC	oral mucosa	56,79	89,56	60,96	4,00	4,00	0	1
68	49	1	0	1	1	0	0	2	0	1	OSCC	tongue	16,13	155,00	8,80	31,00	31,00	0	0
69	72	1	1	1	1	0	0	2	0	1	OSCC	maxilla	84,16	145,59	70,18	27,00	27,00	0	0
70	68	2	1	0	2	0	0	2	0	1	OSCC	floor of mouth	1,30	797,73	80,00	17,00	17,00	0	0
71	79	2	0	1	2	0	0	2	0	1	OSCC	oral mucosa	42,53	111,82	249,76	27,00	27,00	0	0
72	69	2	1	1	2	0	0	2	0	1	OSCC	maxilla	4,08	6,53	45,47	33,00	31,00	1	1
73	55	1	0	0	1	2	0	2	0	1	OSCC	floor of mouth	4,61	9,07	5,44	14,00	6,00	1	1
74	65	2	1	1	3	0	0	2	0	1	OSCC	tongue	52,97	107,62	83,28	27,00	27,00	0	0
75	54	1	1	1	3	2	0	2	0	1	OSCC	tongue	98,88	199,75	178,07	93,00	93,00	0	1
76	44	1	1	0	2	0	0	3	0	1	OSCC	tongue	89,13	249,10	126,73	23,00	5,00	1	0
77	69	2	1	0	4	0	0	2	1	1	OSCC	maxilla	91,43	127,99	1,89	31,00	31,00	0	1
78	73	2	1	0	3	0	0	3	1	1	OSCC	oral mucosa	1,73	2,23	1,79	3,00	3,00	0	1
79	71	1	0	0	1	0	0			0	OPSCC	dist. Oropharynx	91,19	115,18	135,26	1,00	1,00	0	1
80	63	1	1	1	3	1	0			0	OPSCC	base of tongue	7,01	14,69	102,94	10,00	10,00	0	1
81	65	2	1	1	2	0	0	1		0	OPSCC	hard palatine	7,61	28,55	4,41	7,00	7,00	0	1
82	55	1	1	1	1	0	0	3		0	OPSCC	base of tongue	8,64	278,85	24,16	9,00	1,00	1	1
83	55	1	1	1	3	0	0	3		0	OPSCC	hard palatine	20,34	43,77	24,69	4,00	4,00	0	1
84	68	2	1	1	3	2	0	4		0	OPSCC	base of tongue	88,26	176,22	106,51	4,00	4,00	0	1
85	57	1	1	0	2	0	0		0	0	OPSCC	base of tongue	92,00	168,27	32,34	110,00	29,00	1	0
86	62	1	1	1	2	0	0		0	0	OPSCC	base of tongue	1,97	5,06	3,34	17,00	12,00	1	1
87	69	1	1	0	1	0	0		0	0	OPSCC	hard palatine	22,36	37,09	11,00	42,00	42,00	0	1
88	65	2	1	1	2	2	0		0	0	OPSCC	palatine tonsils	75,31	481,87	52,28	3,00	3,00	0	1

89	56	1	1	0	3	3	0		0	0	OPSCC	hard palatine	41,44	63,89	6,76	98,00	98,00	0	1
90	65	2	1	1	4	2	0		0	0	OPSCC	hard palatine	50,12	79,83	51,00	113,00	113,00	0	0
91	70	2	1	1	3	2	0		0	0	OPSCC	palatine tonsils	17,09	48,95	6,18	48,00	48,00	0	0
92	56	2	1	1	1	0	0	1	0	0	OPSCC	hard palatine	33,79	63,04	17,17	126,00	126,00	0	0
93	69	2	0	0	2	1	0	1	0	0	OPSCC	base of tongue	23,21	39,20	2,97	68,00	68,00	0	0
94	54	1	1	0	1	0	0	2	0	0	OPSCC	hard palatine	76,90	152,83	62,29	140,00	140,00	0	0
95	58	1	1	1	2	0	0	2	0	0	OPSCC	hard palatine	90,91	111,54	82,97	98,00	98,00	0	0
96	52	1	1	1	2	0	0	2	0	0	OPSCC	palatine tonsils	92,77	194,37	91,01	112,00	112,00	0	0
97	63	1	1	0	2	0	0	2	0	0	OPSCC	hard palatine	78,13	119,93	78,52	110,00	110,00	0	0
98	54	2	1	0	1	0	0	2	0	0	OPSCC	hard palatine	3,65	74,23	3,81	100,00	100,00	0	0
99	53	2	1	1	2	0	0	2	0	0	OPSCC	palatine tonsils	3,98	36,74	11,29	15,00	15,00	0	1
100	47	2	1	1	2	0	0	2	0	0	OPSCC	base of tongue	11,85	28,01	4,53	41,00	41,00	0	1
101	77	2	1	1	1	0	0	2	0	0	OPSCC	hard palatine	77,55	184,53	57,04	88,00	88,00	0	1
102	61	1	1	1	1	2	0	2	0	0	OPSCC	dist. Oropharynx	88,41	105,36	96,23	1,00	1,00	0	1
103	52	2	1	1	1	2	0	2	0	0	OPSCC	hard palatine	3,75	6,87	,98	126,00	126,00	0	0
104	75	1	0	1	1	1	0	2	0	0	OPSCC	base of tongue	92,49	375,91	69,34	16,00	16,00	0	1
105	73	1	1	1	1	2	0	2	0	0	OPSCC	palatine tonsils	13,48	18,19	7,04	12,00	12,00	0	1
106	71	1	1	1	2	2	0	2	0	0	OPSCC	palatine tonsils	35,03	42,97	40,64	8,00	8,00	0	1
107	72	2	1	1	3	0	0	2	0	0	OPSCC	dist. Oropharynx	2,46	10,67	3,39	37,00	19,00	1	1
108	55	1	1	1	3	2	0	2	0	0	OPSCC	base of tongue	2,20	19,35	1,29	131,00	71,00	1	0
109	61	1	1	1	3	2	0	2	0	0	OPSCC	palatine tonsils	59,06	128,01	61,65	47,00	47,00	0	1
110	64	1	1	1	3	2	0	2	0	0	OPSCC	base of tongue	2,10	4,30	2,20	78,00	78,00	0	1
111	58	2	1	1	1	0	0	3	0	0	OPSCC	palatine tonsils	76,00	246,58	37,81	109,00	109,00	0	0
112	61	2	1	0	1	0	0	3	0	0	OPSCC	hard palatine	1,06	2,09	16,38	140,00	136,00	1	0
113	67	1	1	1	2	0	0	3	0	0	OPSCC	base of tongue	37,35	64,71	5,77	13,00	11,00	1	1
114	51	1	1	1	1	2	0	3	0	0	OPSCC	dist. Oropharynx	27,70	75,56	4,59	,00	,00	0	1
115	56	1	1	1	3	2	0	3	0	0	OPSCC	hard palatine	76,73	81,75	54,70	7,00	7,00	0	1
116	54	2	1	0	4	2	0	3	0	0	OPSCC	dist. Oropharynx	91,97	172,24	79,41	134,00	134,00	0	0
117	70	1	1	0	3	2	0	4	0	0	OPSCC	base of tongue	26,73	53,74	31,19	105,00	105,00	0	0
118	60	1	1	0	2	0	0		1	0	OPSCC	base of tongue	3,48	9,07	1,96	15,00	15,00	0	1

119	65	1	1	0	2	2	0		1	0	OPSCC	palatine tonsils	49,24	236,10	131,50	22,00	22,00	0	1
120	76	2	1	0	3	2	0		1	0	OPSCC	hard palatine	,00	1,07	,55	8,00	8,00	0	1
121	73	1	1	1	1	0	0	1	1	0	OPSCC	hard palatine	20,67	24,02	3,38	63,00	63,00	0	1
122	68	2	1	1	1	0	0	1	1	0	OPSCC	hard palatine	35,37	63,52	48,02	125,00	125,00	0	0
123	49	1	1	1	2	0	0	2	1	0	OPSCC	palatine tonsils	49,00	87,10	63,27	123,00	123,00	0	0
124	82	1	0	1	2	0	0	2	1	0	OPSCC	base of tongue	95,08	162,42	87,57	27,00	16,00	1	1
125	71	2	1	1	1	0	0	2	1	0	OPSCC	hard palatine	43,26	57,71	6,93	47,00	47,00	0	1
126	43	1	1	1	2	2	0	2	1	0	OPSCC	hard palatine	6,44	10,55	6,26	23,00	23,00	0	1
127	54	2	0	0	2	1	0	2	1	0	OPSCC	palatine tonsils	22,15	37,15	9,43	174,00	53,00	1	0
128	61	1	1	1	3	0	0	2	1	0	OPSCC	dist. Oropharynx	80,73	94,50	79,33	11,00	11,00	0	1
129	71	1	1	1	3	0	0	2	1	0	OPSCC	dist. Oropharynx	2,42	40,08	16,27	74,00	74,00	0	1
130	75	1	1	1	3	0	0	2	1	0	OPSCC	base of tongue	24,43	34,60	2,43	80,00	80,00	0	1
131	54	1	1	1	2	0	0	3	1	0	OPSCC	hard palatine	47,79	62,31	16,35	3,00	3,00	0	1
132	62	1	1	1	2	0	0	3	1	0	OPSCC	palatine tonsils	98,18	132,22	71,94	76,00	76,00	0	1
133	71	1	1	1	2	0	0	3	1	0	OPSCC	hard palatine	13,56	52,59	1,56	24,00	24,00	0	1
134	64	1	1	1	2	2	0	3	1	0	OPSCC	palatine tonsils	15,32	34,33	25,92	42,00	32,00	0	1
135	68	1	0	1	1	2	0	3	1	0	OPSCC	dist. Oropharynx	6,31	23,69	,89	20,00	20,00	0	1
136	60	2	1	1	4	0	0	3	1	0	OPSCC	palatine tonsils	,36	,50	,20	12,00	10,00	1	1
137	59	2	1	1	3	1	0	3	1	0	OPSCC	base of tongue	6,39	56,49	30,20	12,00	9,00	1	1
138	58	1	1	1	4	2	0			1	OPSCC	hard palatine	24,04	115,01	32,08	21,00	14,00	1	1
139	64	1	1	1	2	1	0	3		1	OPSCC	palatine tonsils	81,56	127,36	165,29	5,00	5,00	0	1
140	59	1	0	1	2	2	0	3		1	OPSCC	palatine tonsils	14,95	20,53	36,08	24,00	24,00	0	1
141	44	2	0	1	3	0	0	3		1	OPSCC	dist. Oropharynx	,73	1,39	,98	133,00	15,00	1	0
142	68	1	0	1	3	0	0	3		1	OPSCC	hard palatine	16,99	51,46	30,81	13,00	13,00	0	1
143	51	1	0	0	2	0	0	2	0	1	OPSCC	base of tongue	53,71	63,50	141,99	127,00	127,00	0	0
144	55	2	0	1	1	0	0	2	0	1	OPSCC	dist. Oropharynx	2,48	7,24	1,44	14,00	14,00	0	1
145	63	2	1	1	4	2	0	2	0	1	OPSCC	palatine tonsils	57,85	82,07	12,60	105,00	105,00	0	0
146	61	1	1	1	2	0	0	3	0	1	OPSCC	palatine tonsils	30,69	40,89	31,36	133,00	133,00	0	0
147	58	1	0	1	1	1	0	3	0	1	OPSCC	palatine tonsils	15,07	78,35	42,21	114,00	114,00	0	0
148	57	1	1	1	2	1	0	3	0	1	OPSCC	palatine tonsils	60,38	233,28	91,89	88,00	88,00	0	1

149	52	2	1	1	1	2	0	3	0	1	OPSCC	dist. Oropharynx	32,33	149,28	16,45	123,00	123,00	0	0
150	66	1	1	1	2	1	0	3	0	1	OPSCC	palatine tonsils	67,26	86,68	111,32	100,00	100,00	0	0
151	78	1	1	1	1	2	0	3	0	1	OPSCC	palatine tonsils	52,42	84,51	92,53	72,00	2,00	1	0
152	69	1	0	1	2	2	0	4	0	1	OPSCC	palatine tonsils	3,81	57,84	6,44	98,00	98,00	0	0
153	67	1	1	0	4	2	0	4	0	1	OPSCC	hard palatine	86,92	220,11	69,75	71,00	71,00	0	1
154	69	1	1	1	2	0	0	2	1	1	OPSCC	palatine tonsils	60,03	453,94	54,69	111,00	111,00	0	0
155	62	1	1	0	1	2	0	2	1	1	OPSCC	dist. Oropharynx	97,88	151,47	88,36	16,00	16,00	0	1
156	51	2	1	1	1	1	0	2	1	1	OPSCC	palatine tonsils	42,94	68,46	21,36	114,00	114,00	0	0
157	56	2	1	0	1	2	0	2	1	1	OPSCC	palatine tonsils	68,40	96,67	68,26	96,00	96,00	0	0
158	48	1	1	1	2	2	0	3	1	1	OPSCC	palatine tonsils	51,35	119,75	96,59	100,00	100,00	0	0
159	75	1	0	1	3	2	0	3	1	1	OPSCC	base of tongue	3,78	5,87	7,24	58,00	58,00	0	0
160	49	1	1	1	1	2	0	4	1	1	OPSCC	base of tongue	50,39	110,88	54,71	113,00	113,00	0	0
161	49	2	1	0	2	2	0	4	1	1	OPSCC	palatine tonsils	39,17	286,69	56,44	20,00	20,00	0	1
